# Massive Hemoptysis in Previously Treated Pulmonary Tuberculosis: A Case Series

**DOI:** 10.7759/cureus.27882

**Published:** 2022-08-11

**Authors:** Saba Iqbal, Mahmoud Nassar, Ravali Kondaveeti, Tracey O'Brien, Raheel S Siddiqui, Ricardo Lopez

**Affiliations:** 1 Internal Medicine, Icahn School of Medicine at Mount Sinai/New York City Health and Hospitals Queens Hospital Center, New York, USA; 2 Internal Medicine, Icahn school of Medicine at Mount Sinai/New York City Health and Hospitals Queens Hospital Center, New York, USA

**Keywords:** pulmonary, tb, transcatheter arterial embolization, case-series, tuberculosis, massive hemoptysis

## Abstract

Hemoptysis in tuberculosis (TB) is associated with parenchymal distortion and vascular complications linked to prior pulmonary TB. Massive hemoptysis is defined as the expectoration of large volumes of blood. Massive hemoptysis can lead to high morbidity and mortality rates due to hemodynamic instability and airway compromise. In this case series, we present two cases with massive hemoptysis caused by the rupture of the bronchial artery, which achieved hemostasis after fluoroscopy-guided arterial embolization. This series highlights the multiple etiologies of hemoptysis in patients with post-pulmonary TB destruction and the need for various diagnostic and therapeutic modalities. Hemoptysis in patients with prior pulmonary TB can be massive and life-threatening. Timely diagnosis, accurate modality to isolate the source, and appropriate intervention could potentially prevent further lethal complications.

## Introduction

Hemoptysis in tuberculosis (TB) is related to parenchymal distortion and vascular complications associated with prior pulmonary TB. Massive hemoptysis occurs when a large volume of blood is expectorated within 24 hours (between 100 mL and 1,000 mL). Massive hemoptysis can result in high morbidity and mortality rates from hemodynamic instability and airway compromise [1]. In this series, we present two cases with massive hemoptysis from the rupture of the bronchial artery, which achieved hemostasis after fluoroscopy-guided arterial embolization. It highlights the multiple etiologies of hemoptysis in patients with post-pulmonary TB destruction and the need for employing various diagnostic and therapeutic modalities.

## Case presentation

Case 1

**Figure 1:**
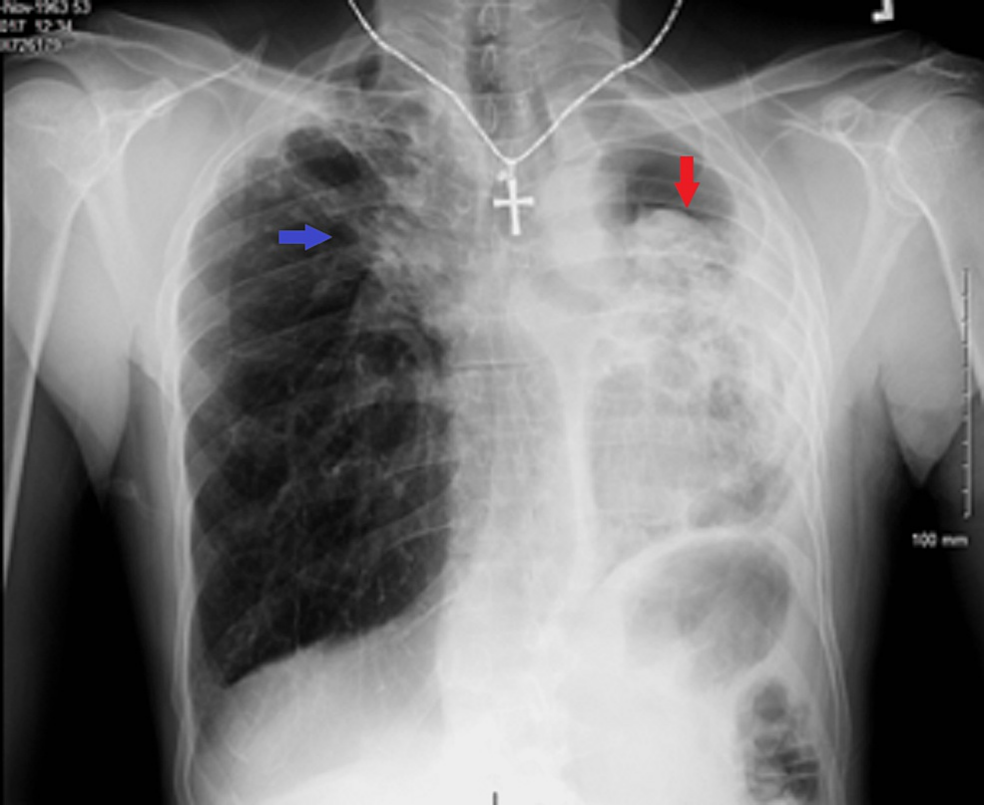
Chest X-ray The image showed extensive parenchymal opacities in the left lung field, associated with left tracheal shift and left-sided pleural thickening (red arrow), and ill-defined reticular opacities in the right upper lobe (blue arrow)

A 56-year-old male immigrant from Guyana presented to the ER with complaints of blood in the sputum and shortness of breath. The patient complained of a productive cough with yellow sputum for about one year, and since the previous day, he had noticed bloody sputum and shortness of breath. The patient was an active smoker with a 76-pack-year smoking habit, and a prior history of active pulmonary TB in Guyana treated with multiple medications for a long but unknown duration with residual lung damage. The physical examination showed left lung crackles. Chest X-ray showed extensive parenchymal opacities in the left lung field and ill-defined reticular opacities in the right upper lobe (RUL) (Figure 1).

**Figure 2:**
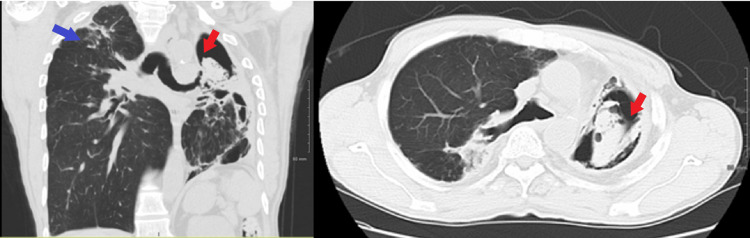
CT chest The images showed a left upper lung zone cavitary lesion with complex amorphous fluid layering dependently, which could represent debris, blood products, or a combination of both (red arrow). Bronchiectasis and architectural distortion in the remainder of the left lung with pleural thickening were seen. Similar findings were observed in the right upper lobe (blue arrow) CT: computed tomography

CT angiography of the chest showed left-sided bronchiectasis, pleural thickening, and left upper lobe cavitation (Figure 2). The patient was admitted to rule out reactivation of active TB and for appropriate management of hemoptysis. Inflammatory markers remained negative, and sputum stains showed no acid-fast bacillus. More than 100 ml of blood was expectorated with cough in a day, and the patient required packed red blood cell transfusions due to a drop in hemoglobin (Hgb).

**Figure 3:**
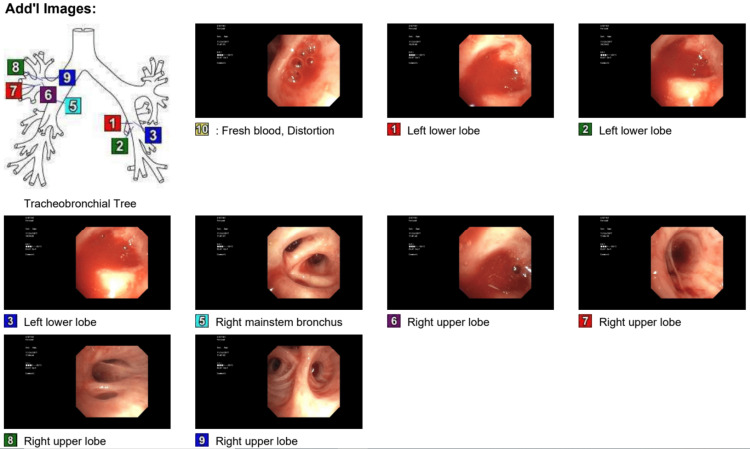
Bronchoscopic pictures of posterior segment RUL, anterior medial LLL, lateral basal segment LLL, and posterior basal segment LLL RUL: right upper lobe; LLL: left lower lobe

The patient's hospital course was also complicated by cardiac arrest and required cardiopulmonary resuscitation for less than two minutes before achieving spontaneous circulation. The patient was then intubated and transferred to the medical ICU. Bedside flexible bronchoscopy showed oozing blood originating in the RUL and left lower lobe (LLL) (Figure 3).

**Figure 4:**
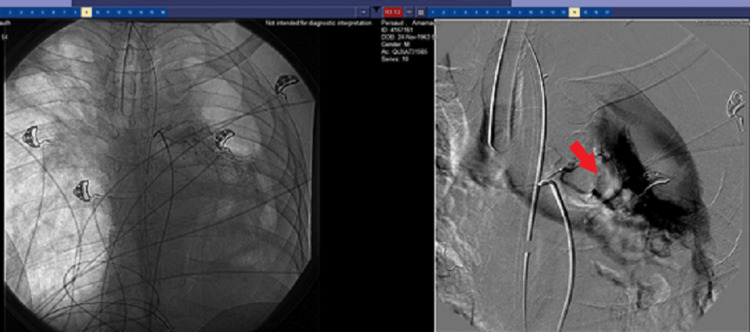
Bilateral bronchial artery embolization Abnormal-appearing left bronchial artery with extravasation of blood (red arrow). No extravasation of blood was noted in the right bronchial artery territory

Under fluoroscopic guidance, the arteriogram showed an abnormal left bronchial artery with extravasation of blood. No extravasation of blood was noted in the right bronchial arterial territory (Figure 4). Embolization was successfully performed in both right and left bronchial arteries due to clinical evidence of bleeding in both the right upper lung lobe and left lower lung lobe on bronchoscopy. The patient tolerated the procedure well and was later discharged home. The patient did not develop a recurrence of hemoptysis more than two years after discharge.

Case 2

**Figure 5:**
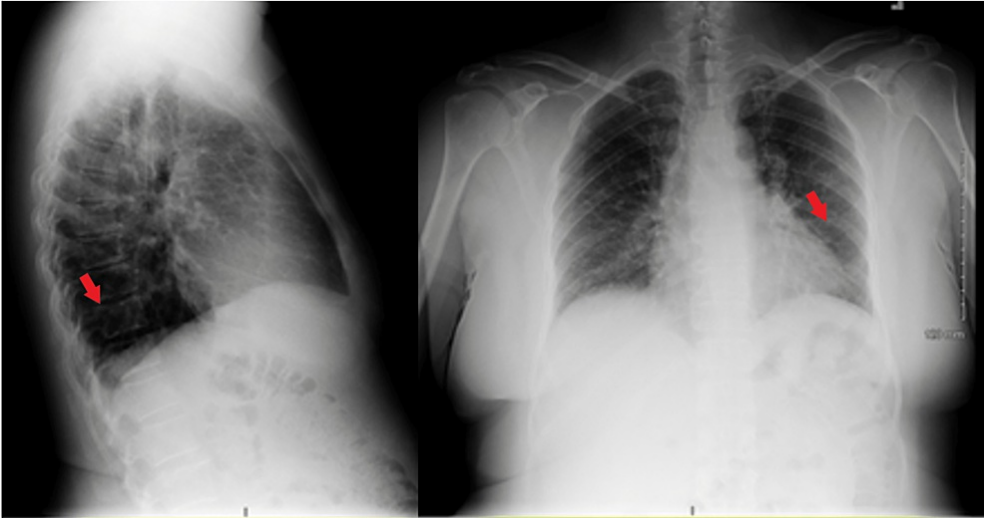
Chest X-ray The images showed coarse, linear opacities at the left lung base (red arrow), mild interstitial prominences at bilateral mid and lower lung fields, left basilar discoid atelectasis, or a scar

A 53-year-old immigrant woman from Guatemala presented to the ER with a one-day history of cough with massive hemoptysis. The patient did not have fever, chills, night sweats, or weight loss, and denied any recent travel history. The patient had a history of active pulmonary TB in Guatemala more than 20 years ago, initially presenting with hemodynamically stable hemoptysis, and she had received treatment with two pills and one injection for a year. The patient again received treatment in the United States about 17 years ago with six months of rifampin, isoniazid, pyrazinamide, and ethambutol (RIPE). Chest X-ray showed coarse, linear opacities at the left lung base, mild interstitial prominences at bilateral mid and lower lung fields, left basilar discoid atelectasis, or a scar (Figure 5).

**Figure 6:**
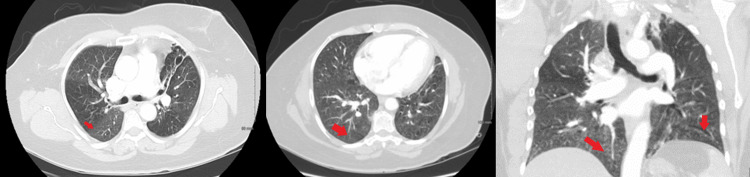
CTA chest The images showed tree-in-bud opacities in RLL, RML, and likely LLL, bilateral fibrosis consistent with prior tuberculosis (red arrow) CTA: computed tomography angiography; RLL: right lower lobe; RML: right middle lobe; LLL: left lower lobe

CTA chest showed bronchiectasis and fibrosis in the upper lobes of both lungs. Reticulonodular nodules were seen in the right middle and lower lung lobe and left lower lung lobe (Figure 6). Inflammatory markers were negative, and sputum stains showed no acid-fast bacillus. Empiric antibiotics and IV hydration were given.

**Figure 7:**
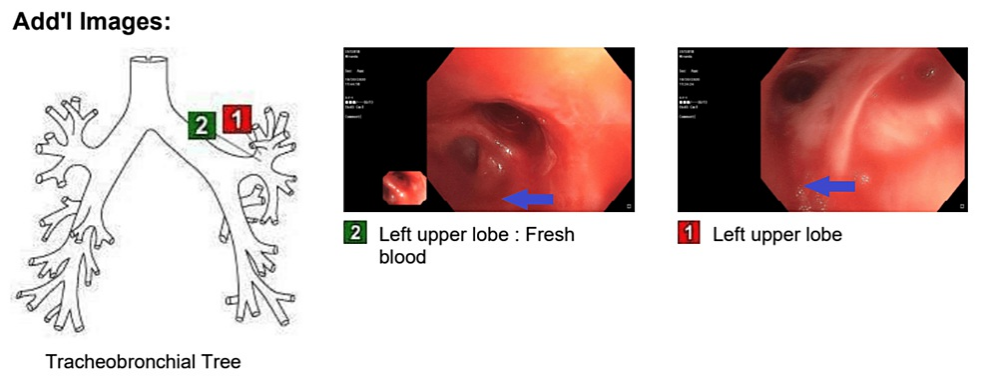
Bronchoscopy showed fresh blood found in the anterior segment of the LUL (blue arrow) LUL: left upper lobe

Flexible bronchoscopy identified the source of bleeding in the left upper lobe anterior segment (Figure 7). Blood was suctioned off, and lavage was done with cold saline followed by epinephrine instillation. Fluid obtained by lavage was also negative for infections. Under fluoroscopic guidance, the arteriogram showed an extremely hypertrophied and tortuous left bronchial artery with an area of abnormal blush. Bleeding was stopped by embolization of the left bronchial artery. The hemoptysis was resolved, and the patient was observed for 48 hours before discharge. At two months of follow-up, the patient remained symptom-free.

## Discussion

Hemoptysis in the presence of active or previously treated TB can be due to multiple etiologies such as bronchiectasis, TB reactivation, scar carcinoma, aspergillomas, broncholiths, lung cavity, and pseudoaneurysms [2,3]. Post-TB cavitations and bronchiectasis changes in the lung could lead to irreversible damage to the lung parenchyma and its vasculature. Rasmussen aneurysm is a pseudoaneurysm that forms adjacent to a tuberculous cavity. It entails a herniation of the vessel into the cavity and weakening of the walls of the vessels, which results in rupture of the vessel leading to massive hemoptysis [3]. In our cases described here, patients with a history of TB treated with rifampin, isoniazid, pyrazinamide, and ethambutol presented with massive hemoptysis causing hemodynamic instability.

Although hemoptysis is commonly associated with active TB infection, it can also be seen after completing medical therapy for pulmonary TB. Of note, promptly identifying the source of hemoptysis will help direct the treatment. The source of bleeding could be the pulmonary or bronchial arteries, the most common being the bronchial arteries. It is difficult to differentiate the source of hemoptysis based on clinical symptoms alone. Contrast-enhanced multidetector CT angiography (MDCTA) can help identify the source of bleeding [4]. Flexible fiberoptic bronchoscopy is the initial procedure of choice for the localization of the bleeding and to achieve hemostasis [5]. If urgent fiberoptic bronchoscopy is not available, then thoracic CTA can be used as a diagnostic modality to guide therapeutic intervention. Optimal diagnostic sensitivity can be achieved by the combination of CTA and bronchoscopy [6].

Emergency endovascular techniques like interventional radiology-guided arterial embolization represent the first-line treatment for massive hemoptysis [7]. Both the cases described here underwent bronchoscopy initially to locate the source of bleeding and later proceeded with IR arterial embolization to achieve hemostasis. Surgical excision is recommended if radiological intervention is not readily available or if there is a considerable destructive process in the lung due to prior infection [8].

## Conclusions

Hemoptysis in patients with prior pulmonary TB can be massive and life-threatening. Timely diagnosis, adopting a proper modality to isolate the source, and appropriate intervention could potentially save patients from further lethal complications.
